# Editorial: Moving the mind, thinking the body: new insights on the mind-body connection from the neuroscience of movement, sports, arts, yoga, and meditation

**DOI:** 10.3389/fnhum.2024.1376909

**Published:** 2024-02-07

**Authors:** Maria Elide Vanutelli, Bernhard Hommel, Alice Cancer

**Affiliations:** ^1^Department of Psychology, University of Milan-Bicocca, Milan, Italy; ^2^Department of Psychology, Shandong Normal University, Jinan, China; ^3^Department of Psychology, Università Cattolica del Sacro Cuore, Milan, Italy

**Keywords:** embodied cognition, embodiment, neuroscience, movement, sports, aesthetics, yoga, mind-body

Humans have long pondered the connection between mind and body, both inside and outside the scientific community. Cartesian dualism, so ingrained in Western culture, has for years prevented any attempt to set the question differently. In the last 50 years, this boundary begun to blur as integrative approaches, occasionally drawing inspiration from older traditions from the Eastern world (Varela et al., 2017), arose in cognitive science. Through them, the fundamental role of the body in learning processes was emphasized, as well as that of psychological factors in movement. Although widespread at the theoretical level, this approach still struggles to gain a foothold from the scientific point of view. In fact, the methodologies to investigate the manifestations of body and mind seem to be not always compatible, but probably only apparently so. On these assumptions, the idea of the present Research Topic was born. “Moving the Mind, Thinking the Body” brings together the most recent contributions addressing the mind-body problem.

Starting from the protective role of physical activity in neurological rehabilitation, Maeneja et al. conducted a clinical trial on post-stroke patients, comparing the cognitive recovery effects of aerobic exercise with those of a dual-task intervention. Results revealed significant improvements in both attention and cognitive functioning after the physical exercise intervention, and that such improvements were stronger as compared to the dual-task intervention, suggesting that physical exercise is the best option for enhancing cognitive recovery in post-stroke.

Physical activity can also promote social cognition. Effective interactions with others rely on anticipatory representations of their actions. To investigate the role of sport expertise in predicting the outcome of a competitor's action, Cancer et al. implemented an experimental paradigm for testing the recognition of the opponent's intentions in sailing. The authors hypothesized that individuals with prior sailing motor expertise would be more accurate in predicting the outcome of the observed sailing maneuvers, as compared to non-sportsmen and sportsmen who practiced a different sport, namely tennis. Sailors outperformed the other groups, likely due to the activation of domain specific motor representations of the movements observed. On the contrary, the activation of irrelevant motor patterns led tennis players to have the worst performance in the sailor's intention recognition, thus adding evidence to the implication of the mirror neuron system in action anticipation.

Considering the complexity of human functioning, cognition is not the only dimension positively affected by physical activity. Psychological wellbeing and emotional functioning have largely been studied in relation to sport and movement performance. Dance was reported to reduce the level of stress, anxiety, and depression and enhance mood, confidence, and energy (e.g., Salihu et al., 2021). In their attempt to further the understanding of the relation between dance practice and mental health, Kulshreshtha et al. conducted a study on a sample of Kathak dancers. Kathak is a classical dance form, prevalent in Northern India, characterized by fast dynamic footwork that acts as a medium to release anger and tension. Compared to a sample of non-dancers, the Kathan performers showed lower stress and reduced depressive and anxiety symptoms. Based on these findings, the authors highlighted the potential use of Kathan dance as a protective intervention for the risk of developing depression and anxiety.

Given the benefits of movement on psychological wellbeing, providing breaks for physical exercise during working hours is a common practice in China and other countries to reduce stress and improve cognitive performance (Zheng et al., 2013). One of the main components of these types of exercises are breathing techniques, like Baduanjin, which are known to reduce stress and anxiety (e.g., Cho et al., 2016). In the attempt to compare the effect of different breathing techniques on relaxation (measured as muscle tension) and executive functions, Liang et al. conducted a within-subject electromyographic study. Mindful breathing reduced muscle tension, while slow breathing was specifically effective in improving the performance of male participants on an inhibition task. These outcomes were supported by an improved respiration efficiency, as tested through the modulation of respiration rate and oxygen saturation.

A context that is potentially associated with stress and anxiety is public speaking. García-Monge et al. assessed the efficacy of a specific program in reducing the effect of public speaking anxiety over psychophysiological responses and self-report measures of anxiety. The Corp-Oral Program was designed by readapting previous techniques within an embodiment framework. During a simulated public speaking test, participants were invited to redirect their anxiety by using body awareness, embodied message techniques, simulation, embodied visualization, body transformation, and gesture enhancement. Results revealed decreased anxiety scores and physiological response. This effect was also reflected in EEG data, with frontal alpha asymmetry indicating less avoidance attitudes.

Another innovative area for exploring the advantages of the mind-body connection is aesthetic experiences, which involve embodied mechanisms, such as the simulation of actions, emotions, and corporeal sensations, including a sensorimotor component (Freedberg and Gallese, 2007). Ardizzi et al. engaged 5-years-old children in free-hands manipulations with sand or clay. Before and after the manipulations, children were invited to rate artistic artifacts made of different sculpting materials for beauty, smoothness, and darkness. Results revealed that the more the children had the opportunity to manipulate, the more positive were the values they expressed about the artworks made of the same sculpting material. The authors thus concluded that aesthetic experience is specifically linked to its sensorimotor component and rooted on bodily experience.

Finally, Lynn and Basso's methodological study concerned the creation and validation of a self-report scale measuring the multifaceted ways in which movement affects the individual at a physical and psychological level (Multidimensional Impacts of Movement Scale, MIMS). The scale was built by combining ancient yogic tradition with current neuroscientific knowledge and includes subscales representing the body, energy, mind, intuition, and contentment. To test its psychometric properties with specific regard to validity testing, the authors asked participants to complete the MIMS after their practice, 2 weeks later. The analyses proved MIMS to be a valid and reliable tool to measure the impact of movement on a wide range of wellbeing variables.

To conclude, we believe that this Research Topic gathered various innovative contributions to further the mind-body reciprocal positive effects. These studies provided valuable implications for the development of health promotion interventions, without a net distinction between medical, psychological, or social wellbeing. We hope that this valuable collection will be helpful for researchers in various fields, by kicking off a research line that explicitly sheds more and more light on the connection between mind and body.

## Author contributions

MV: Writing—original draft, Writing—review & editing, Conceptualization. BH: Writing—review & editing. AC: Writing—original draft, Writing—review & editing.
